# 

**Published:** 1906-12-15

**Authors:** 


### You have often needed a Book

That would aid you in emergency. That would help you to recollect some point you had forgotten or about which you had a doubt.

That would prove of assistance in difficulty.

That would convey information clearly and concisely on Diseases; their Symptoms and Nursing Treatment.

### The Nurse's "Enquire Within"

is a Pocket Encyclopædia of valuable nursing information alphabetically arranged, and

### Fills a Want that has long been felt by Nurses everywhere. It is bound in limp cloth boards with rounded corners, and its size and weight will admit of its being carried conveniently in the apron pocket without injury thereto.

### The NURSES' PRONOUNCING DICTIONARY OF MEDICAL TERMS AND NURSING TREATMENT.

**2/-** post free. **2/-** post free.

COMPILED BY

**HONNOR MORTEN.**

NEW EDITION.

Revised and Edited by MARY I. BURDETT.

NEW AND REVISED EDITION.

### SURGICAL INSTRUMENTS AND APPLIANCES.

**1/6** net, **1/6** net,

**1/8** post free. **1/8** post free.

An Illustrated and Classified List, with Explanatory Notes.

CONTAINING A NEW CHAPTER ON

THE CARE AND STERILIZATION OF SURGICAL INSTRUMENTS.

BY

**HAROLD BURROWS, M.B.(Lond.), B.S., F.R.C.S.**

**Write for Catalogue of Nursing Manuals, which will be sent post free on application.**

### The Scientific Press, Ltd.

Publishers of Nursing Text-Books, Charts, and Case-Books of all Descriptions.

28 & 29 SOUTHAMPTON STREET, STRAND, LONDON, W.C.

